# Supplementary material for: Dysfunctions of Neutrophils in the Peripheral Blood of Children with Cystic Fibrosis
Source: Biomedicines. 2023 Jun 15;11(6):1725. doi: 10.3390/biomedicines11061725 (PMC10296301; doi:10.3390/biomedicines11061725)
Supplement: Supplementary file 1 [file biomedicines-11-01725-s001.zip › biomedicines-2430571-supplementary.pdf]

Supplementary Materials

Table S1. Antibodies used in phenotypic analysis of neutrophils.

Antibody	Dilution	Manufacturer
CD11b	1:200	Biolegend
CD15	1:50	Biolegend
CD44	1:100	Biolegend
CD16	1:100	Biolegend
CD11c	1:100	Biolegend
CD206	1:100	ThermoFisher Scientific
PDL-1	1:100	Biolegend
Dectin 1	1:100	Biolegend
TLR2	1:100	Biolegend
CCR5	1:100	Biolegend
ILT3	1:50	ThermoFisher Scientific
ILT4	1:50	Biolegend
IDO-1	1:200	R&D Systems
TGF-β	1:50	R&D Systems
IL-10	1:50	Biolegend
IL-6	1:50	Biolegend
IL-4	1:50	Biolegend
MPO	1:100	ThermoFisher Scientific
CD14	1:100	Biolegend
CD86	1:100	ThermoFisher Scientific
NLRP3	1:100	ThermoFisher Scientific
IL-17A	1:30	R&D Systems
TNF-α	1:50	Biolegend
IL-33	1:50	ThermoFisher Scientific
HLA-DR	1:100	Biolegend
CD54	1:100	Biolegend
FASL	1:50	Biolegend

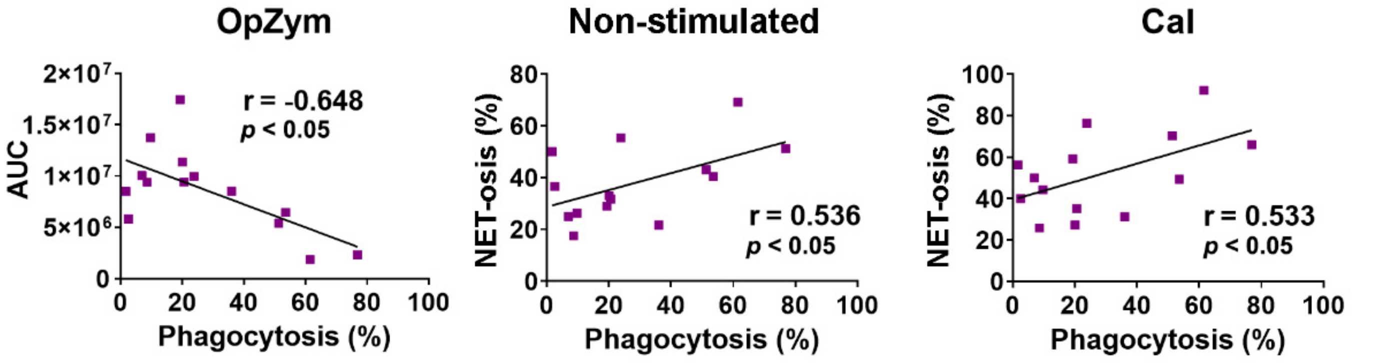

Figure S1. Correlation between phagocytosis and reactive oxygen species (ROS) production and NET-osis in neutrophils of control children. AUC-Area under the curve; OpZym-Opsonized zymosan; CaI-Calcium ionophore.

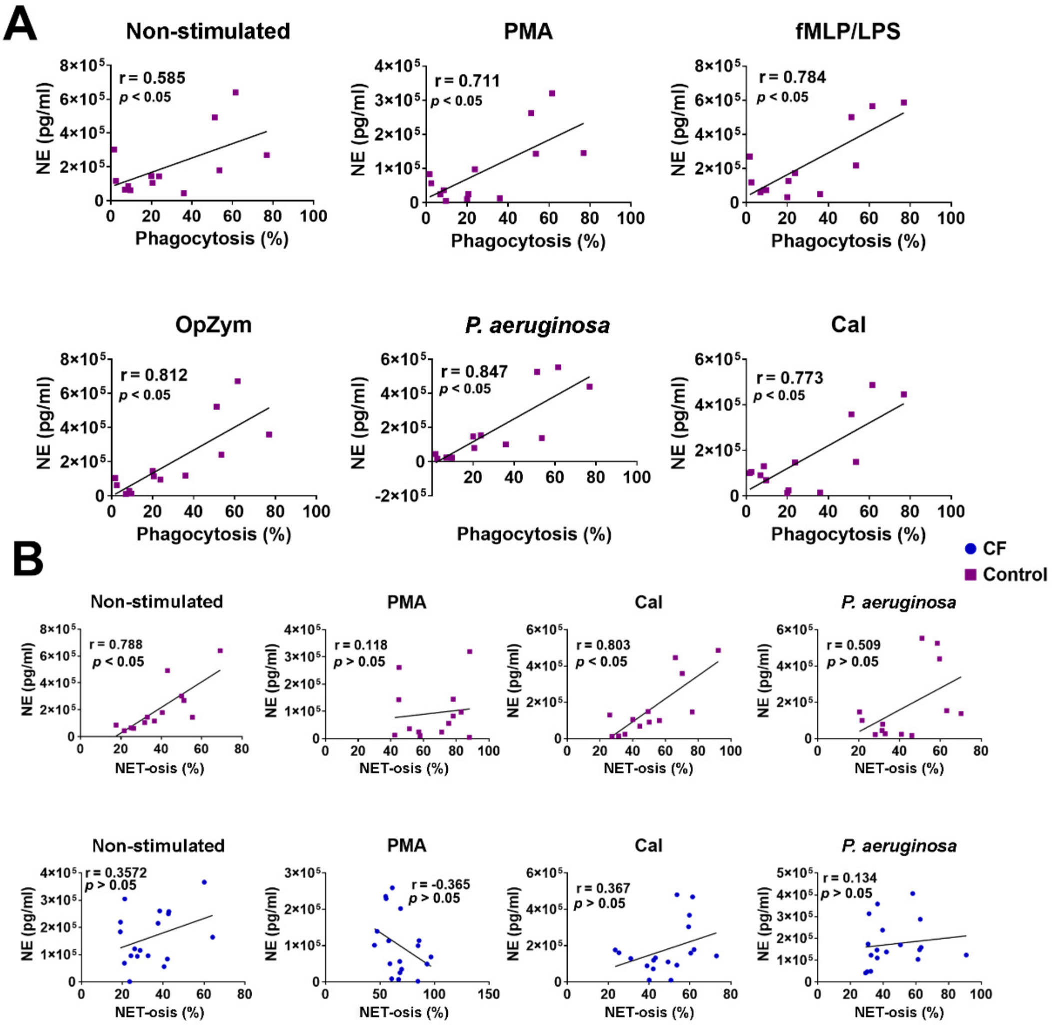

Figure S2. (**A**) Correlation between phagocytosis of neutrophils of healthy children and neutrophil elastase (NE) levels in culture supernatants; (**B**) Correlation between NET-osis of neutrophils from cystic fibrosis (CF) or control children and NE levels in culture supernatants. PMA-phorbol myristate acetate; fMLP/LPS-*N*-formyl-methionyl-leucyl-phenylalanine and lipopolysaccharide; CaI-calcium ionophore; OpZym-Opsonized zymosan.

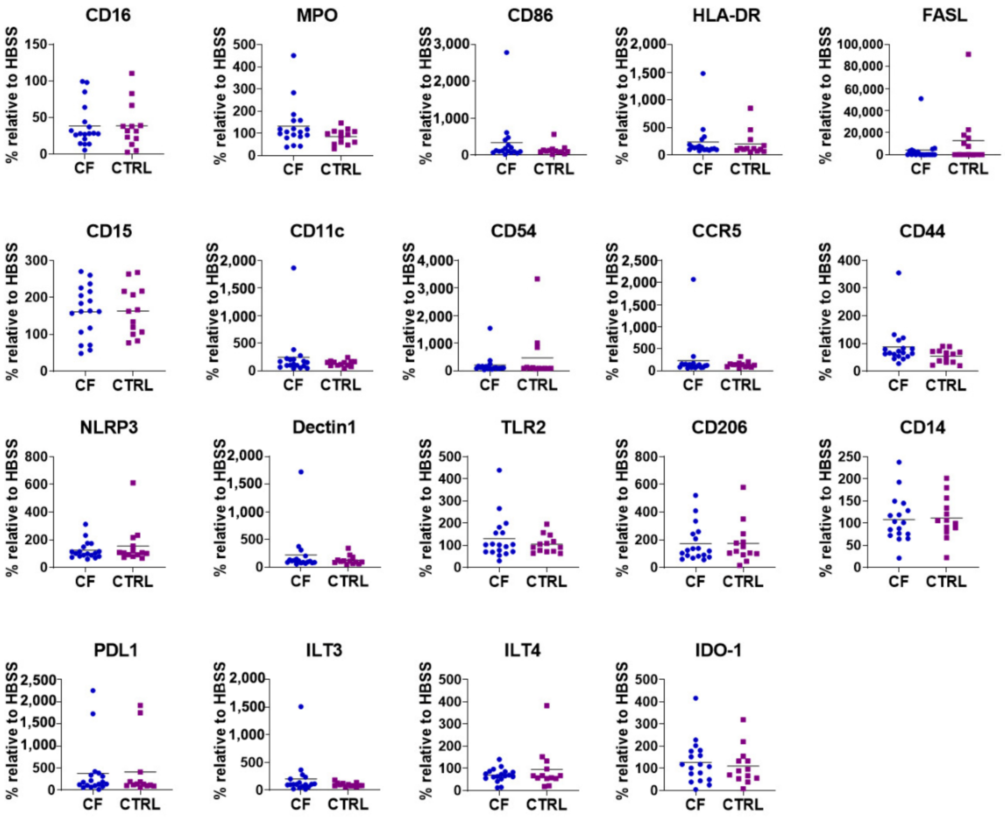

Figure S3. Phenotypic characteristics of neutrophils from cystic fibrosis (CF) and control children. Results are presented as the percentage change in the mean fluorescence intensity (MFI) of stimulated neutrophils compared to non-stimulated cells (basal fluorescence of neutrophils in HBSS medium).

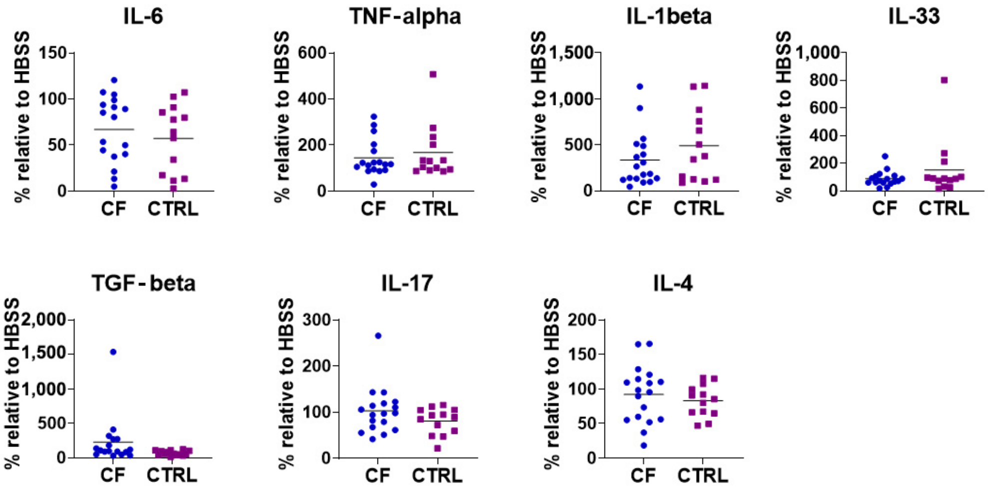

Figure S4. Intracellular expression of cytokines in cystic fibrosis (CF) and control neutrophils. Results are presented as the percentage change in the mean fluorescence intensity (MFI) of stimulated neutrophils compared to non-stimulated cells (basal fluorescence of neutrophils in HBSS medium).
